# High-frequency ultrasound combined with shear wave elastography to evaluate the efficacy of lymphaticovenular anastomosis in patients with secondary lymphedema

**DOI:** 10.3389/fsurg.2025.1578436

**Published:** 2025-08-29

**Authors:** Haige Yu, Shuo Zhang, Haohui Zhu, Xiao Ding, Chengkai Wu, Chenjing Xu, Yan Song

**Affiliations:** ^1^ Department of Ultrasonography, Henan Provincial People’s Hospital, Zhengzhou, China; ^2^ Graduate School, Xinxiang Medical University, Xinxiang, Henan, China

**Keywords:** lymphedema, high-frequency ultrasound, shear wave elastography, lymphatic venous anastomosis, subcutaneous echo-free space

## Abstract

**Purpose:**

High-frequency ultrasound combined with shear wave elastography (SWE) was used to evaluate the efficacy of lymphaticovenular anastomosis (LVA) in treating secondary lymphedema.

**Materials and methods:**

This study included 40 patients with secondary lymphedema who underwent LVA at the Department of Vascular Surgery, Henan Provincial People's Hospital, from October 2023 to October 2024. Limb circumference measurements, bioelectrical impedance analysis (BIA), and high-frequency ultrasound combined with SWE were conducted before and after treatment. Changes in subcutaneous echo-free space (SEFS) grading and elastic parameters were analyzed pre- and post-treatment.

**Results:**

Following LVA, the percentage of excess volume (PEV) and the extracellular water/total body water (ECW/TBW) ratio in the affected limb significantly decreased (*P* < 0.001). SEFS scores of the subcutaneous tissue also showed statistically significant changes (*P* < 0.001). Additionally, the shear wave velocity (SWV) of the dermis decreased, while that of the subcutaneous tissue layer increased, both with statistical significance (*P* < 0.001). The difference in SEFS scores between pre- and post-treatment was statistically significant (*P* < 0.001), supporting the finding that SWV decreased in the dermis and increased in the subcutaneous tissue layer (*P* < 0.001).

**Conclusion:**

The reduction in SEFS in the affected limb after LVA, along with the decreased SWV in the dermis and increased SWV in the subcutaneous tissue layer, suggests that LVA effectively reduces lymphatic fluid retention across all tissue layers. This provides novel ultrasonographic evidence for assessing and refining treatment efficacy and follow-up.

## 1 Introduction

Lymphedema is a chronic condition caused by defects or injuries in the lymphatic system, leading to protein and fluid accumulation in the interstitial spaces of tissues due to impaired lymphatic fluid return. This results in limb edema, and as the disease progresses, patients may experience inflammation, tissue proliferation, and fibrosis, which are disabling and difficult to treat. These complications severely impact daily activities and reduce quality of life (1). Lymphedema is categorized based on etiology into primary and secondary forms. Secondary lymphedema (SL) commonly occurs as a complication following radical surgery for clinical tumors, with a prevalence of 14%–40% (2, 3). Given the variability in disease progression, early diagnosis and intervention are considered the most effective strategies.The primary clinical methods for assessing lymphedema treatment efficacy include limb circumference (LC) measurements, bioelectrical impedance analysis (BIA), and other diagnostic tests. LC measurement is widely recognized for its simplicity and broad clinical use (4). BIA, a newer technique, accurately measures extracellular water in both limbs, reflecting the degree of edema (5). However, LC and BIA results mainly indicate overall limb changes post-surgery. In contrast, high-frequency ultrasound provides detailed visualization of subcutaneous tissue echogenicity, especially the distribution of subcutaneous echo-free space (SEFS). The quantity of echo-free space (EFS) intuitively reflects the fluid load and soft tissue swelling. Additionally, shear wave elastography (SWE) quantitatively assesses the hardness of the dermis and subcutaneous tissue layer. The combination of these modalities offers a more precise evaluation of edema in the affected limb (6, 7). Lymphaticovenular anastomosis (LVA) is a minimally invasive procedure that connects lymphatic vessels to subcutaneous superficial veins, creating a new lymphatic return pathway. This technique directs excess lymphatic fluid in the edematous limb into the venous system for therapeutic purposes (8). This study aimed to investigate the clinical value of high-frequency ultrasound combined with SWE in assessing the therapeutic effect of LVA in patients with secondary lymphedema.It is worth noting that achieving successful outcomes with LVA in the lower limbs can be more challenging compared to the upper limbs due to factors such as gravity, longer lymphatic pathways, and potentially higher tissue pressures. Therefore, evaluating the efficacy of LVA specifically in lower limb lymphedema using objective imaging modalities like HFUS-SWE is of significant clinical interest.

## 2 Materials and methods

### 2.1 Patient selection

This study included forty patients with secondary lower limb lymphedema who underwent lymphaticovenous anastomosis (LVA) at the Vascular Surgery Department of Henan Provincial People's Hospital from October 2023 to October 2024. Among the participants, 34 were female and 6 were male, with ages ranging from 28 to 70 years (mean age: 51.25 ± 11.43 years). The duration of limb edema ranged from 1 to 120 months, with a median duration of 23.84 months (interquartile range: 20.88 months),indicating a relatively shorter duration of lymphedema in this cohort compared to some long-standing cases reported in the literature. Patients’ body mass index (BMI) ranged from 18.75 to 39.46 kg/m^2^, with a mean of 26.87 ± 3.72 kg/m^2^. Inclusion criteria were as follows: (1) unilateral lower limb swelling following radical tumor surgery, tumor radiation therapy, or local lymph node dissection; (2) lymphoscintigraphy confirming impaired lymphatic drainage in the affected limb, characterized by findings such as delayed tracer transit, dermal backflow, and/or abnormal tracer distribution patterns, alongside evidence of radionuclide accumulation in the inguinal lymph nodes on the contralateral (healthy) side serving as an internal reference; bioimpedance analysis (BIA) showing increased extracellular water in the affected limb; and MRI findings suggesting subcutaneous tissue thickening and fluid accumulation, confirming unilateral limb lymphedema; (3) no history ofprior LVA treatment; (4) complete clinical data and voluntary participation in the study. Exclusion criteria included: (1) postoperative infection or thrombosis in the affected limb; (2) tumor recurrence during treatment. This study received approval from the Ethics Committee of Henan Provincial People's Hospital, and all patients provided informed consent.

### 2.2 Treatment and assessment

#### 2.2.1 LVA

Patients diagnosed with lower limb lymphedema after admission, who met surgical eligibility criteria, underwent preoperative ultrasound localization of functional lymphatic vessels. This was complemented by intraoperative indocyanine green lymphography to identify lymphatic vessels with good drainage function. Lymphatic vessel anastomosis was then performed under general anesthesia by experienced vascular surgeons with senior titles.

#### 2.2.2 Measurement of limb circumference

**Figure 1:**
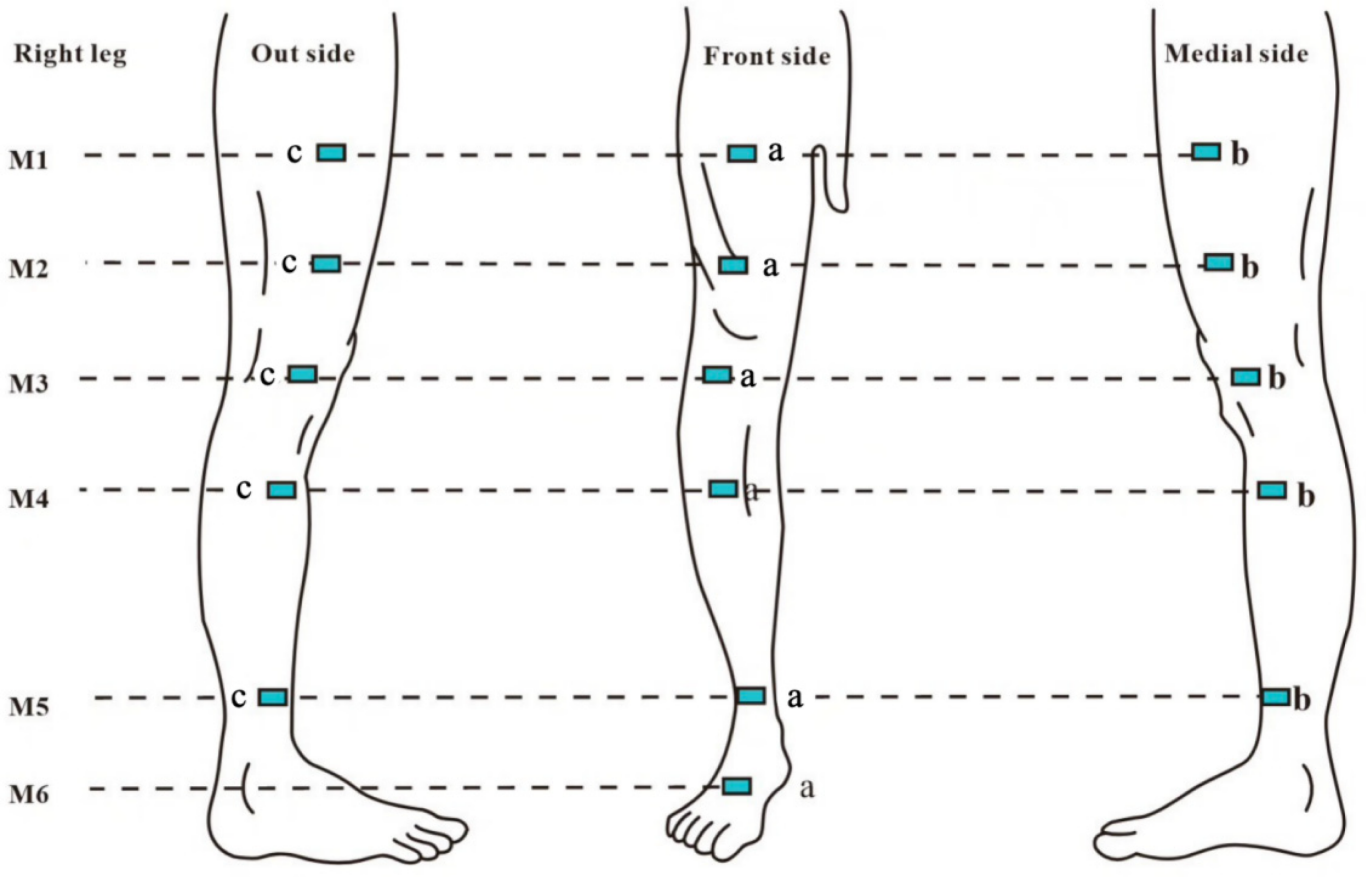
M1: 10 cm above the superior border of the patella; M2: at the level of the superior border of the patella; M3: 10 cm below the superior border of the patella; M4: midpoint of the calf (midpoint of the line connecting the tip of the patella and the ankle transverse stripe); M5: at the level of the ankle transverse stripe; M6: at the dorsum of the foot, specifically the midpoint of the line between the ankle transverse stripe and the tips ofthe webbing between the 2nd and 3rd toes; a: intersection points between each horizontal plane and the line connecting the midpoint of the groin to the tip of the patella, as well as the line connecting the tip of the patella to the transverse crease of the ankle joint (anterior lateral point); b: medial 1/4 circumference (medial point); c: lateral 1/4 circumference (lateral point).

Patients were positioned supine, and circumference was measured using a soft ruler with 1 mm accuracy. Each plane was measured three times consecutively, and the average value was recorded. The circumference of each plane was divided into four equal parts, with measurement points marked as shown in Figure 1. Measurements were repeated one week post-operation, and markings were reapplied. Circumferences (C) and spacings (H) were used in the truncated cone volume formula: V = H × (C1 + C1 × C2 + C2)/12π, where C1 is the circumference of the proximal end and C2 is the circumference of the distal end of the adjacent segment. The percentage of excess volume (PEV) was calculated as the difference between the volumes of the affected and healthy limbs, expressed as PEV = (affected limb volume—healthy limb volume)/healthy limb volume × 100%. Improvement in affected limb volume before and after treatment was denoted as *Δ*PEV.

#### 2.2.3 Bioelectrical impedance analysis

The InBody770 Multi-Frequency Segmented Bioelectrical Impedance Body Composition Tester (Bysbys Medical Devices Co., Ltd., Jinshan, Shanghai, China) was used to assess body composition in a quiet state, following strict operational guidelines. Indicators measured included total body water (TBW), extracellular water (ECW), extracellular water ratio (ECW/TBW), and bioelectrical impedance analysis (BIA).

#### 2.2.4 High-frequency ultrasound and shear-wave elastography

**Figure 2:**
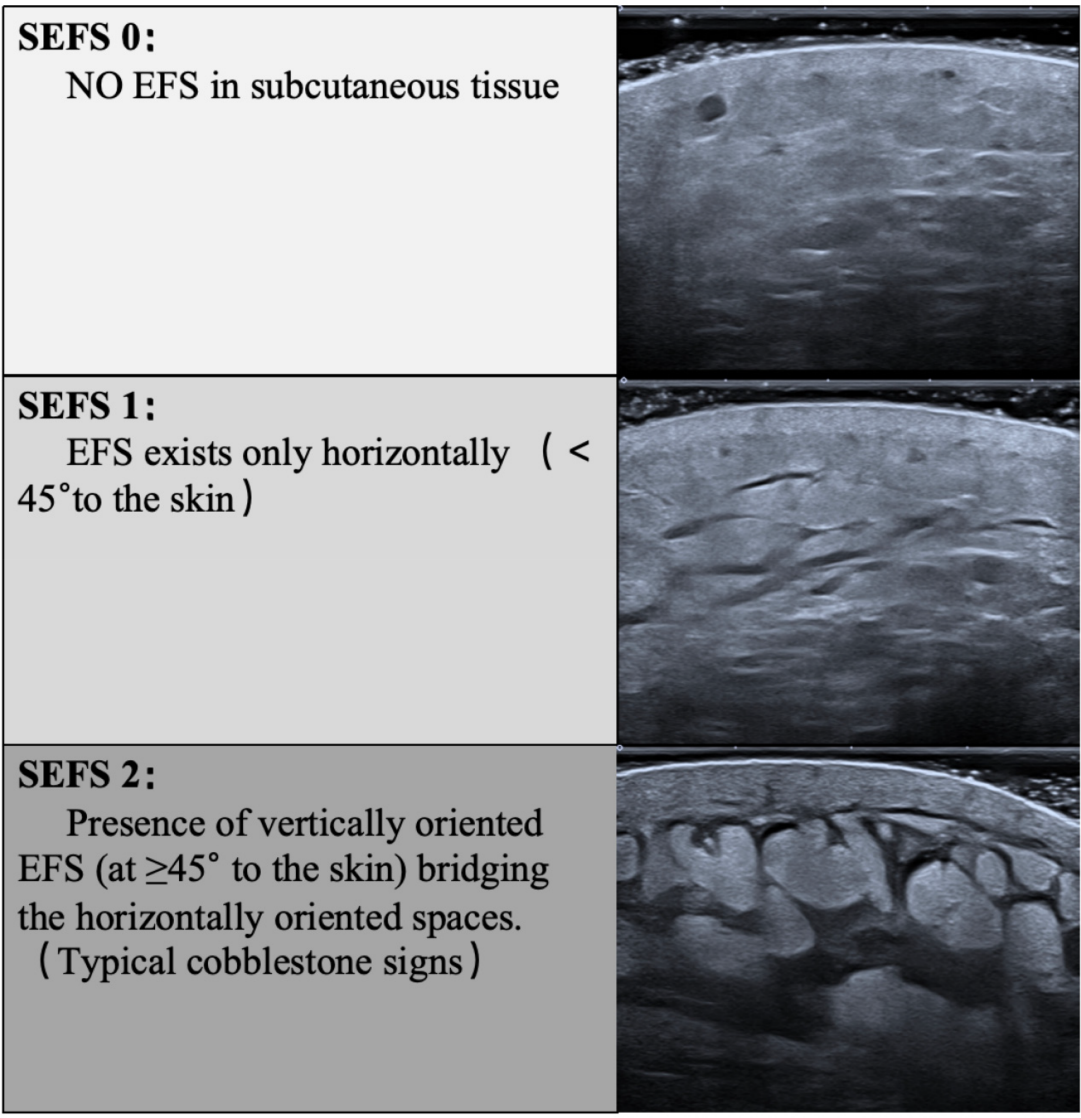
Criteria of subcutaneous echo-free space (SEFS) grade.

**Figure 3:**
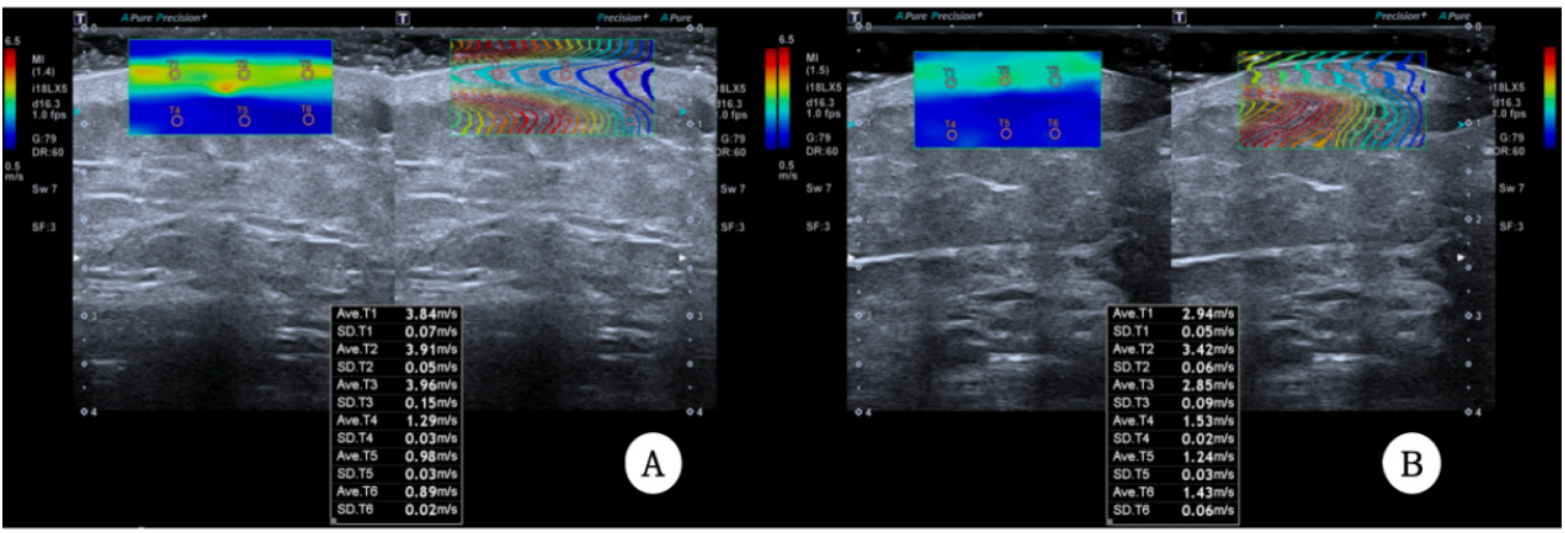
**(A)** and **(B)** show preoperative and postoperative elastography in SWE mode.

A Canon Aplio i900 color Doppler ultrasound diagnostic machine was used, featuring an 18 MHz PLI-1205BX probe equipped with Shear Wave Elastography (SWE) mode. The method synchronized control acquisition between the affected and healthy sides. Based on the SEFS grading standard Figure 2), each measurement point was graded and scored (grade 0 = 0 points, grade 1 = 1 point, grade 2 = 2 points), yielding a total score. The SWE mode was then activated, and the probe was held perpendicularly to the skin surface in a suspended position. Once the waveform stabilized, three regions of interest (ROIs) were placed consecutively within the dermis, and parameters within these ROI regions were automatically calculated to obtain Shear Wave Velocity (SWV) EQs for both the dermis and subcutaneous tissue layers, including the SWV Emean of the subcutaneous tissue layer (Figure 3). Measurements were repeated one week post-surgery. The improvement rate of each ultrasound parameter before and after treatment was calculated using the formula: (before treatment—after treatment)/before treatment × 100%.

#### 2.2.5 Patient-reported outcome measures (PROMs)

Patients completed the validated Lymphedema Quality of Life (LYMQOL) questionnaire preoperatively and at 1-week post-LVA. LYMQOL assesses four domains: symptoms, body image, physical function, and mood (each scored 1–4,lower scores indicate better QoL). The overall QoL score (range 1– 10) and domain-specific changes were analyzed.

### 2.3 Statistical analysis

IBM SPSS version 26.0 was used for data analysis. Continuous variables were assessed for normality; those conforming to a normal distribution were reported as mean ± standard deviation, while non-normally distributed variables were expressed as median and interquartile range (IQR). Comparisons between groups before and after treatment were conducted using repeated measures ANOVA. Correlation analyses were performed using the Pearson test for normally distributed data and the Spearman test for non-normally distributed data. Semiquantitative data were also analyzed using the Spearman test. Intra- and inter-operator reproducibility were evaluated using the Bland-Altman plot method.

## 3 Results

### 3.1 Status of LVA treatment

The number of LVA anastomotic branches in the affected limbs of all patients ranged from 7 to 18, with a mean of 11.45 ± 2.41.

### 3.2 Patient-Reported outcomes

Patients reported significant improvements in quality of life after LVA treatment. Medianoverall QoL scores (LYMQOL) increased from 5 (IQR: 2) to 8 (IQR: 1) on a 10-point scale (*P* < 0.001), indicating a transition from “moderate” to “good” perceived recovery. All symptomdomains showed marked relief (Table 4): Physical function: Score decreased 43% (3.5 ± 0.6 →2.0 ± 0.5, *P* < 0.001) Limb discomfort: Symptom score decreased 44% (3.2 ± 0.4 → 1.8 ± 0.3,*P* < 0.001) Body image concerns: Reduced 41% (3.7 ± 0.3 → 2.2 ± 0.4, *P* < 0.001).

### 3.3 Objective clinical improvements

Consistent with subjective reports, technical metrics confirmed edema reduction:Limb volume:Excess volume (PEV) decreased by 53% (43.03% → 20.50%, *P* < 0.001) Tissue fluid retention:Extracellular water ratio (ECW/TBW) reduced by 3.2% (42.73 ± 1.70 → 41.35 ± 1.49, *P* < 0.001) Skin softening: Dermal stiffness (SWV) decreased 13%–24% across measurement points (e.g., thigh:2.71→2.16 m/s, *P* < 0.001) Swelling resolution: Subcutaneous fluid pockets (SEFS score) dropped 64% (22→8 points, *P* < 0.001) (Tables 1–3 detail all measurements).

**Table 1:** Comparison of PEV and ECW/TBW before and after treatment of affected limbs.

Groups	Before treatment (%)	After treatment (%)	value of statistics	*p*-Value
PEV	43.03（32.01）	20.50（22.01）	t = 251.53	P<0.001
ECW/TBW	42.73 ± 1.70	41.35 ± 1.49	t = 77.12	P<0.001

Note: PEV, percentage of excess volume; ECW/TBW, extracellular water/total body water.

**Table 2:** Comparison of dermal shear wave velocity and subcutaneous tissue layer shear wave velocity before and after surgery.

Groups	SWV in the dermis （m/sec）				SWV in the subcutaneous tissue（m/sec）			
	Before treatment	After treatment	value of statistics	*p*-Value	Before treatment	After treatment	value of statistics	*p*-Value
M1a	2.71（0.74）	2.16（0.56）	z = 72.44	<0.001	1.72 ± 0.18	1.91 ± 0.22	t = 81.05	<0.001
M1b	2.67（0.72）	2.20（0.51）	z = 69.16	<0.001	1.88 ± 0.31	1.95 ± 0.52	t = 65.94	<0.001
M1c	2.87 ± 0.47	2.34 ± 0.52	t = 113.21	<0.001	1.70 ± 0.31	1.92 ± 0.22	t = 88.96	<0.001
M2a	2.94（0.59）	2.53（0.50）	z = 45.33	<0.001	1.80 ± 0.46	1.98 ± 0.30	t = 85.25	<0.02
M2b	2.97（0.75）	2.63（0.74）	z = 115.32	<0.001	1.77（0.40）	2.06（0.43）	z = 57.83	<0.001
M2c	3.06（1.03）	2.54（0.81）	z = 136.83	<0.001	1.78（1.59, 2.06）	2.27（1.99, 2.46）	z = 89.65	<0.001
M3a	4.05 ± 0.69	3.34 ± 0.58	t = 110.72	<0.001	1.85 ± 0.31	2.26 ± 0.71	t = 77.05	<0.001
M3b	4.03 ± 0.75	3.07 ± 0.76	t = 53.70	<0.001	1.88 ± 0.44	2.28 ± 0.53	t = 67.91	<0.001
M3c	4.02 ± 0.75	3.14 ± 0.66	t = 44.91	<0.001	1.81（0.36）	2.21（0.51）	z = 102.47	<0.001
M4a	4.32 ± 0.57	3.45 ± 0.55	t = 117.62	<0.001	1.92 ± 0.41	2.37 ± 0.40	t = 83.66	<0.001
M4b	4.29 ± 0.25	3.44 ± 0.62	t = 93.20	<0.001	1.79（0.48）	2.35（0.52）	z = 79.13	<0.002
M4c	4.07 ± 0.62	3.35 ± 0.61	t = 78.19	<0.001	1.77（1.53, 1.94）	2.06（1.82, 2.28）	z = 83.65	<0.001
M5a	4.17 ± 0.76	3.31 ± 0.66	t = 69.97	<0.001	1.81 ± 0.41	2.43 ± 0.52	t = 51.11	<0.001
M5b	4.28 ± 0.75	3.45 ± 0.68	t = 28.13	<0.001	1.77 ± 0.39	2.04 ± 0.38	t = 74.28	<0.001
M5c	4.24 ± 0.63	3.59 ± 0.58	t = 73.28	<0.001	1.85 ± 0.29	2.03 ± 0.37	t = 110.82	<0.001
M6	3.90 ± 0.82	3.14 ± 0.61	t = 72.50	<0.001	1.84 ± 0.33	2.43 ± 0.75	t = 71.69	<0.001

Note: a, Anterior lateral point; b, medial point; c, lateral point.

**Table 3:** Comparison of SEFS scores before and after surgery.

Groups	Before treatment（score）	After treatment（score）	value of statistics	*p*-Value
SEFS	22.00（11.00）	8.00（6.75）	t = 81.23	P<0.001

Note: SEFS, subcutaneous echo-free space.

### 3.4 Changes in PROMs

Postoperative LYMQOL scores showed significant improvement in all domains (*P* < 0.001, Table 4). The median overall QoL score increased from 5 (IQR: 2) to 8 (IQR: 1),enhanced patient-perceived recovery.

**Table 4:** Patient-reported symptom improvement after LVA.

Domain	Pre-LVA	Post-LVA	value of statistics	*P*-value
Symptoms	3.2 ± 0.4	1.8 ± 0.3	t = 92.30	<0.001
Physical Function	3.5 ± 0.6	2.0 ± 0.5	t = 112.35	<0.001
Body Image	3.7 ± 0.3	2.2 ± 0.4	t = 120.11	<0.001
Mood	3.0 ± 0.5	1.9 ± 0.3	t = 89.44	<0.001
Overall QoL	5 (2)	8 (1)	z = 79.32	<0.001

Note: Data presented as mean ± SD or median (IQR).

### 3.5 Correlation of post-treatment improvement rate with *Δ*PEV and *Δ***ECW/TBW for each elasticity parameter and SEFS score**

**Figure 4:**
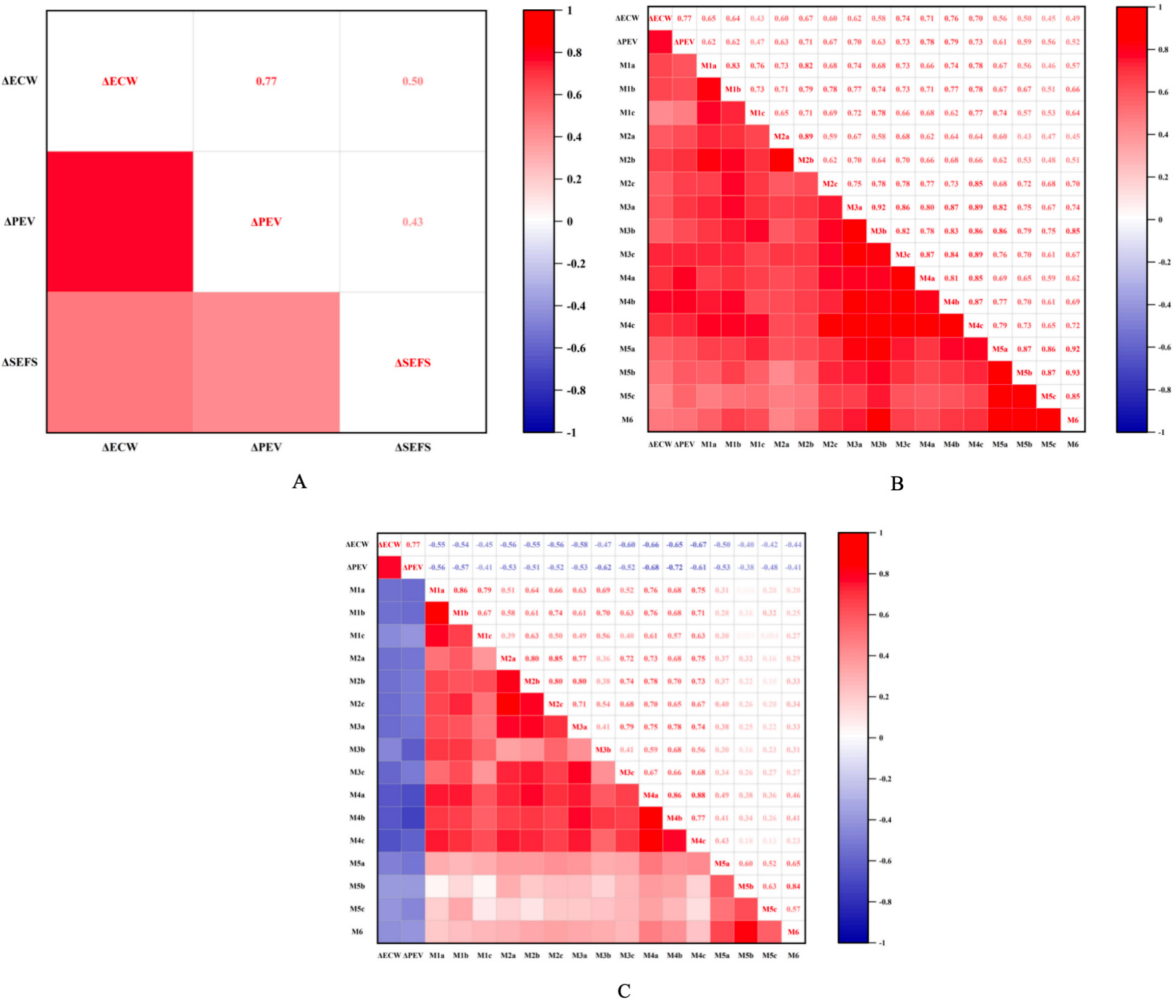
**(A)** Illustrates the correlation between *Δ*SEFS, *Δ*PEV, and *Δ*ECW/TBW. **(B)** Depicts the correlation between the improvement rate of shear wave velocity in the dermis layer and both *Δ*ECW and *Δ*ECW/TBW at each measurement point. **(C)** Shows the correlation between the improvement rate of shear wave velocity in the subcutaneous tissue layer and *Δ*ECW and *Δ*ECW/TBW at each point. Here, *Δ*ECW refers to *Δ*ECW/TBW, denoting the change in the extracellular water ratio before and after surgery. Additionally, *Δ*PEV represents the change in excess volume ratio before and after surgery, while *Δ*SEFS indicates the proportion of the change in SEFS scores ofpatients before and after surgery.

The improvement rate of dermal shear wave velocity (SWV) was positively correlated with the change in peripheral edema volume (*Δ*PEV) at all measurement points (all *P* < 0.05), showing the strongest correlation at the calf midpoint plane (all r > 0.7, all *P* < 0.01). Additionally, the dermal SWV improvement rate was positively correlated with the change in extracellular water to total body water ratio (*Δ*ECW/TBW) (all *P* < 0.05), with correlation coefficients at the calf segment higher than those at the thigh segment. Conversely, the subcutaneous tissue SWV improvement rate demonstrated a negative correlation with both *Δ*PEV and *Δ*ECW/TBW, with stronger correlations observed in planes above and below the knee joint, as well as at the mid-calf plane (all r < −0.47, all *P* < 0.01). Furthermore, the change in subcutaneous extracellular fluid status (*Δ*SEFS) was positively correlated with both *Δ*PEV and *Δ*ECW/TBW (all r > 0.43, all *P* < 0.05) (Figure 4).

### 3.6 Consistency test

**Figure 5:**
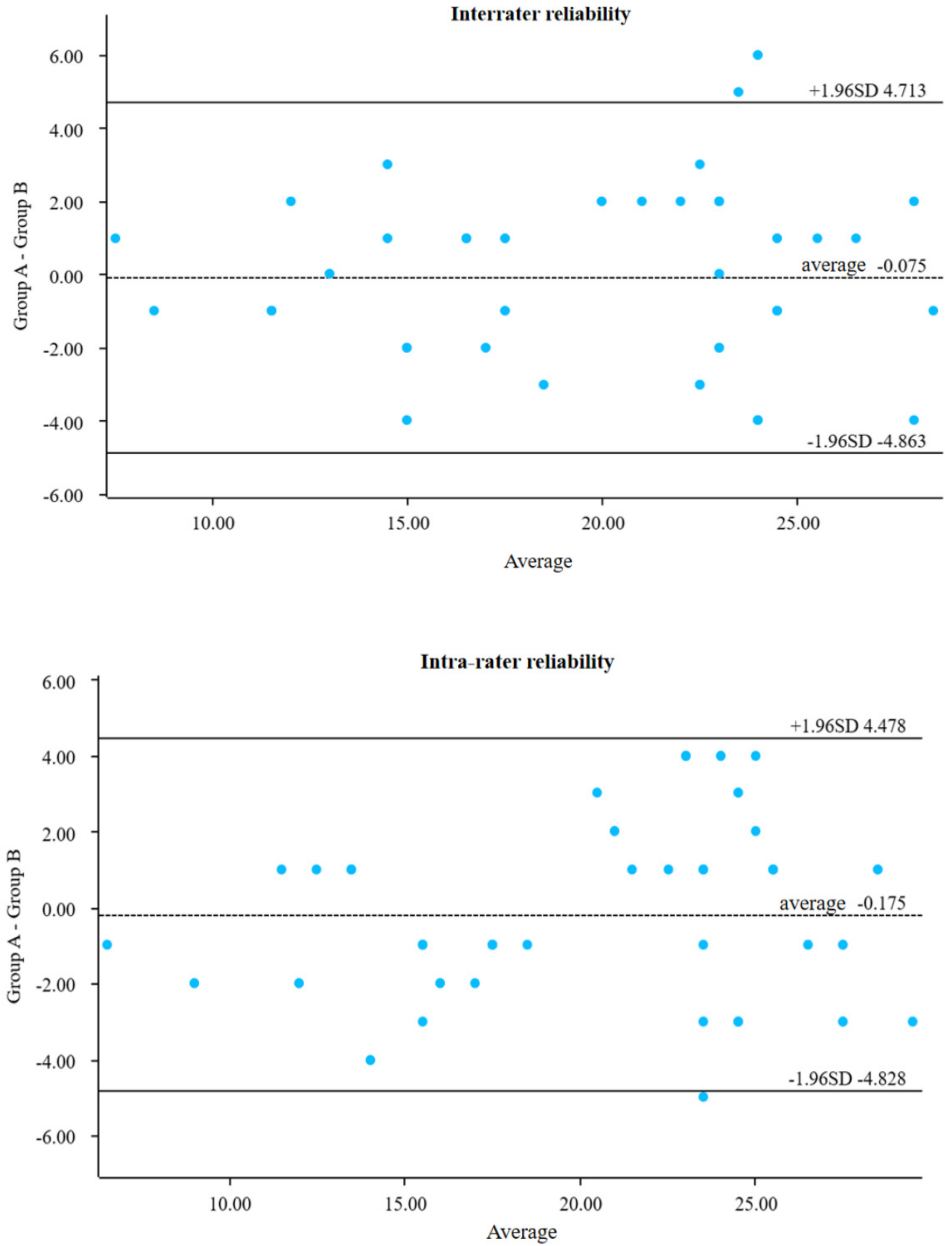
Consistency test bland-altman plot.

Ten patients were selected for preoperative and postoperative assessment. The same so nographer evaluated SEFS scores at each measurement point, and a senior attending sonog rapher repeated the assessment to conduct interrater and intrarater reproducibility tests. The results showed good agreement, with intra-collector intervals of 95% CI: −4.863 to 4.713 and inter-collector intervals of 95% CI: −4.828 to 4.478 (Figure 5).

## 4 Discussion

Lower limb volume increases and mobility decreases in patients with lymphedema, severely impacting their daily lives (9). Lymphatic vessel anastomosis (LVA) restores lymphatic circulation by reestablishing lymphatic return pathways, reducing fluid overload in the affected limb, decreasing fat deposition and tissue fibrosis, and improving clinical symptoms (10). Consequently, accurately evaluating the efficacy of this surgical intervention is essential in the clinical management of lymphedema.Beyond quantitative tissue changes, LVA providedclinically meaningful benefits: 80% of patients reported improved mobility and reduced discomfort as early as 1-week postoperatively. This rapid symptom relief—validated by both LYMQOL scores and objective reductions in dermal stiffness/fluid retention—demonstrates LVA's dual biophysical markers and patient well-being.

High-frequency ultrasound combined with shear wave elastography (SWE) technology allows precise measurement of changes in shear wave velocity (SWV) within the dermis and subcutaneous tissue layers of the limb before and after surgery (11, 12), and assesses the reduction in skin elasticity and firmness score (SEFS) in the subcutaneous tissue layers. In this study, patients treated with LVA exhibited a significant reduction in limb volume, an improvement in swelling, and a decrease in extracellular water, as indicated by postoperative bioelectrical impedance analysis (BIA), consistent with previous findings (13). The postoperative reduction in dermal SWV, increase in subcutaneous tissue SWV, and decrease in SEFS in the subcutaneous tissue layer may be attributed to decreased extracellular water content in limb tissues, particularly in the subcutaneous tissues, due to enhanced lymphatic fluid return. This process alleviates the fluid load on subcutaneous soft tissues, resulting in reduced skin tension and stiffness (14, 15). Kotaro et al. (16) also demonstrated that the affected limb improved after transient lymphatic drainage edema, particularly in the calf segment, with increased skin tissue strain indicating skin softening, consistent with the findings ofthis study.The significant improvements observed in limb volume (PEV), extracellular water (ECW/TBW), subcutaneous fluid distribution (SEFS), and tissue stiffness (SWV) following LVA in our cohort are encouraging, particularly considering the recognized challenges associated with treating lower limb lymphedema. Factors such as gravity-dependent fluid accumulation and the complex anatomy of lower limb lymphatics often make outcomes less predictable compared to upper limb cases. Our findings suggest that HFUS-SWE provides sensitive indicators of successful physiological changes even in this challenging anatomical location.

The reduction in affected limb volume following LVA involves not only fluid dynamics but may also be influenced by additional factors, such as decreased muscle content due to prolonged immobilization, increased fat content, and other changes in body composition post-surgery. However, *Δ*PEV and *Δ*ECW/TBW effectively reflect the reduction in extracellular water content of the affected limb post-surgery, minimizing the impact of other body composition changes. This study demonstrated a strong correlation between *Δ*PEV and *Δ*ECW/TBW, indicating that lymphatic pathway repair and improved lymphatic fluid return following LVA reduced extracellular water within the subcutaneous tissues and limb swelling. This outcome is consistent with post-LVA assessments using BIA alone (17). Toshima et al. (18) showed that increased SEFS, in the context of lower extremity lymphedema and limb swelling, was closely associated with elevated extracellular water percentage. In this study, changes in SEFS scores of the affected limb before and after treatment correlated well with ΔPEV and ΔECW/TBW.

Correlation analysis of dermal SWV and the improvement rate of subcutaneous tissue SWV with ΔPEV and ΔECW/TBW of the affected limb revealed that most measurement points for dermal SWV improvement rate had a strong positive correlation with ΔPEV and ΔECW/TBW. Conversely, the improvement rate of subcutaneous tissue SWV showed a negative correlation with these variables, particularly at points with thicker subcutaneous tissue, which had higher correlation coefficients. Notably, correlation coefficients for the dorsum of the foot, midpoint of the foot, and ankle points were higher than those for other points. The coefficients were especially elevated at locations with thicker subcutaneous tissue, while those for the dorsum of the foot and ankle points were lower due to thinner subcutaneous tissue. Although the subcutaneous tissue layer was thicker at thigh segment points, the correlation was less pronounced, possibly due to factors like gravity or impaired lymphatic valve function in peripheral lymphatic vessels, leading to more severe lymphedema in the calf segment (19). Consequently, the calf segment may accumulate more lymphatic fluid, with more severe histopathology, making it a focal point in lymphedema treatment of the lower limbs.

Importantly, the technical improvements (reduced SEFS, dermal SWV, and ECW/TBW) aligned with patient-reported outcomes. The significant enhancement in LYMQOL particularly in physical function and symptoms—confirms that LVA not only resolves biophysical markers but also alleviates daily discomfort and improves psychosocial well-being. This synergy between objective metrics and PROMs underscores LVA ‘ s holistic efficacy. (20) While PROMs were collected at 1 week post-LVA to capture early changes, longer-term follow-up (e.g., 6 – 12months) is needed to evaluate sustained QoL improvements.

The combination of ultrasonography, circumferential measurements, and BIA provides a comprehensive assessment of changes across all tissue layers of the affected limb before and after LVA treatment. However, this study has limitations: the patient sample was predominantly composed of individuals with stage II and III lymphedema, with limited research on patients in subclinical stages. Secondly, the mean BMI of our cohort (26.87 kg/m^2^) was relatively low. Higher BMI is a known risk factor for lymphedema severity and can complicate surgical outcomes and imaging interpretation due to increased adipose tissue. The favorable results observed here may, in part, reflect the lower adiposity in our patients. Caution is warranted when extrapolating these findings to populations with higher average BMIs, as tissue composition and fluid dynamics may differ. Thirdly, the median duration of lymphedema in our study was approximately 2 years. Chronic lymphedema (e.g., >5 years) often involves more advanced tissue changes, including significant fibrosis and adipose deposition. These chronic changes might be less reversible with LVA and could potentially manifest differently on HFUS-SWE (e.g., persistent stiffness despite volume reduction). Our findings primarily reflect the response in patients with moderate disease duration. The applicability of HFUS-SWE parameters to assess LVA efficacy in very long-standing lymphedema warrants further investigation.Futuremulti-centerstudies should stratify outcomes by BMI to explore this relationship further.

## 5 Conclusion

The application of high-frequency ultrasound combined with shear wave elastography (SWE) provides a detailed evaluation of changes across various tissue levels in the affected limb, both before and after surgical treatment for lymphedema. This approach offers a reliable ultrasound imaging basis for targeted clinical interventions and introduces a novel imaging technique for postoperative follow-up and the development oftherapeutic strategies.

## Data Availability

The original contributions presented in the study are included in the article/Supplementary Material, further inquiries can be directed to the corresponding author.
